# Chlorine Water as an Antiseptic in Operations and Injuries of the Eye

**Published:** 1892-05

**Authors:** Alvin A. Hubbell

**Affiliations:** Göttingen


### CHLORINE WATER AS AN ANTISEPTIC IN OPERATIONS AND INJURIES OF THE EYE.

By SCHMIDT-RIMPLER, of Göttingen.

Translated by ALVIN A. HUBBELL, M. D., from *Annales d'Oculistique.*

The disinfecting power of officinal chlorine water (German Pharmacopeia) has been known a long time, and my experience was published in 1877, in Volume LXX. of *Virchow's Archives.* I made punctures in the cornea of rabbits with an instrument charged with the products of the secretion of chronic blennorrhagic dacryocystitis, which is a potent agent of suppuration. The germs were introduced by the aid of a needle into the puncture in the corneal tissue. Before the inoculation, the infectious liquid was brought under the influence of various disinfecting solutions, and the extent of the antiseptic effect was measured by the absence or modification of the corneal suppuration. The comparison between chlorine water, salicylic acid, phenic acid, thymol, and quinine has resulted in recognizing the chlorine water as the more potent antiseptic. Experiments since made have led to the same result. In placing cultures in contact with disinfectant solutions, Buchholz, Koch and his assistants, Sattler, Weeks, and others, have had occasion to notice the extraordinary antiseptic power of chlorine water. These two series of experiments mutually complete each other. It is not sufficient to ascertain the influence of antiseptics on the development of cultures, but it is necessary also to test their action on the products of the workings of microörganisms, especially on infected structure. The researches of Fischer and Proskauer[1] have shown that humidity, also, of the microörganisms plays an important role. Cultures soaked with water were surely and rapidly killed by the chlorine in relatively thick layers. In inoculating the *staphylococcus pyogenes aureus* into the cornea, I have observed that the sterilization was more rapid in cultures

1. Ueber die Desinfection mit Chlor. und Brom., *Mittheilungen aus dem Kaiserl. Gesundheitsamt*, Vol. II., 1884.

emanating from liquified gelatine than in relatively dry cultures that were at the beginning of their development. The first ordinarily became sterile one minute or less in the chlorine water. It is proper, however, to consider that the intensity of the suppuration provoked by different cultures of *staphylococcus* varies. The influence of the chlorine water on the secretion of the lachrymal sac was also prompt, while that of solutions of bichloride, such as are applied in ophthalmology (1 to 5,000), were very inferior. The same culture of *staphylococcus pyogenes aureus* that became sterile after one minute in the chlorine water remained very virulent after three minutes in the bichloride solution. To render sterile the secretion of purulent inflammation of the lachrymal sac, it was necessary to allow five to ten minutes of contact with the bichloride solution, three to five minutes simply weakening its virulence. The experiments with pus from the lachrymal sac are much more decisive in ocular antisepsis, as it is the microbes from this that are more often the cause of operative suppurations of the cornea, and as the microbes exposed to the influence of antiseptics are in analogous conditions to those that provoke corneal suppurations. Notwithstanding its extraordinary bactericidal power, chlorine water has not been generally applied as an antiseptic in ocular operations.

Previous to the past year I applied it in isolated cases only, where the danger of an infection was imminent; for example, in operations on patients affected with dacryocystitis. Since that time it has served me as an antiseptic for all operations. I believe it proper to publish the observations collected on this subject, and to recommend, strongly, the employment of chlorine water in ophthalmology.

Up to the month of July of this year (1891), I have applied the chlorine water in 125 cases of extraction of cataract. Among the first twenty-five cases operated upon at Marburg there was one of primitive suppuration of the corneal wound. The lips of the wound illy adapted themselves to each other, and an alcoholic delirium necessitated a change of the dressing a short time after the operation. It is quite possible, therefore, that the infection was secondary. The 100 extractions made since then at Göttingen were without incident, as well as other operations to the number of 375, among which were several plastic operations.

The corneal infiltrations, provoked by the sublimate after the instillation of cocaine, do not occur with the application of chlorine water, this, to my mind, constituting a considerable advantage. I will add that cocaine, in several operated upon, has produced extended superficial infiltrations and epithelial lesions that were increased by the manipulations necessary in the evacuation of the cortical remains of the lens. It was the same in the cases where the flap had been everted by the upper lid. These corneal infiltrations, due to the application of sublimate, compromise in an unpleasant manner the success of cataract operations, although preventing, almost absolutely, suppuration. Thus, Alfred Graefe,[1] who has made a very large use of this antiseptic substance, reports : "In 1,074 operated upon for cataract, suppuration occurred only in 0.93 per cent., but in 4.7 per cent. of the cases there was an inferior acuity of vision of one-ninth, on account of corneal infiltrations. The chlorine water prevents the suppurations without injuring the cornea.

There are two special objections to the general employment of chlorine water in traumatisms and operations on the eyes : the fear of too much irritation, and its tendency to undergo decomposition. I am convinced that these are not justified. Far from irritating the wound or conjunctiva, the chlorine water produces less conjunctival secretion than the sublimate. It causes a slight smarting of the eye that is unnoticeable under the cocaine anesthesia. It is hemostatic, and colors the blood cherry-red. In the second place, certain precautions suffice to prevent the chlorine water from decomposing. The flasks, closed by glass or rubber stoppers, should not exceed the capacity of 250 to 500 grammes (eight to sixteen ounces), and their contents excluded as much as possible from the influence of air and light, the two principal agents of its decomposition. I have found that the water in the bottom of a flask, whose contents have served during four weeks in the dispensary, still rendered sterile the secretion of the lachrymal sac. Analysis showed that it contained 0.23 per cent. of chlorine, instead of 0.4 per cent., which represents the proportion of the German pharmacopeia. Two cubic centimeters of chlorine water, which had remained eight days in a flask of 500 grammes, supplied three and a half weeks before, was always active. As long as the liquid presents the characteristic odor of chlorine, it may be applied. A more exact test is obtained by the use of litmus paper, which is bleached by the active chlorine water ; the presence of hydrochloric acid is shown by the litmus paper changing to a reddish tint.

1. *Graefe's Archiv.*, Vol. XXXV., Part 3, 1889.

Concerning the question of price, it is proper to consider that the bactericidal power, so pronounced in the chlorine water, makes the application of large quantities unnecessary; 250 grammes, of which the price is one franc fifty centimes (twenty-seven cents), have sufficed for ten operations.

In plastic operations on the lids, and traumatisms of the eye, the results obtained with this substance have been as satisfying as those obtained in intraocular operations. In cases of corneal suppuration, especially in serpiginous ulcer, irrigations with it, repeated several times a day, have given me very satisfactory results, although operations may still be necessary in grave cases. In these, the operation of Sæmisch seems to me preferable to the application of the galvano-cautery.

The *modus faciendi* employed in operations is the following: The instruments are placed in a solution of phenic acid (1 to 20) for half an hour, and then put in alcohol. They are next immersed in phenic solution (1 to 40), and given to the operator without being dried. I believe them to be more antiseptic when wet, and the contact of the phenic acid with the incision favors, perhaps, primary union. The patient takes a bath before the operation, and the face is cleaned with soap. Shortly before the operation, and before the instillation of cocaine, the lids and their surroundings are washed with sublimate solution, and the conjunctival cul-de-sac and globe with chlorine water. During the instillation of the cocaine, the eye is kept covered with a muslin compress wet with chlorine water. When the patient is laid down, the cul-de-sac and globe are again irrigated with chlorine water, and the lids wiped off with muslin compresses moistened with the water. The irrigation is repeated when the operation is finished. The lids are then covered with muslin compresses wet with chlorine water and cotton sterilized with bichloride, and the whole is fixed by a sterilized bandage. The compresses wet with chlorine water are kept in an enameled jar, covered, to avoid as much as possible the inconveniences of the penetrating odor of the chlorine. The first dressing is kept on four days, when it is renewed in the same manner. If the skin is irritated, the dressing is replaced by a dry one of boracic gauze and sublimated cotton. The wet dressings require some precautions. I have seen abscess of the lids with suppuration of the cervical glands take place under dressings wet repeatedly with bichloride.

The good results obtained with the chlorine water in plastic operations of the lids, healing taking place without any secretion, justifies methodical trials of this antiseptic in other branches of surgery.

